# A Modular Lentiviral and Retroviral Construction System to Rapidly Generate Vectors for Gene Expression and Gene Knockdown *In Vitro* and *In Vivo*

**DOI:** 10.1371/journal.pone.0076279

**Published:** 2013-10-11

**Authors:** Benjamin Geiling, Guillaume Vandal, Ada R. Posner, Angeline de Bruyns, Kendall L. Dutchak, Samantha Garnett, David Dankort

**Affiliations:** Department of Biology, McGill University, Montréal, Quebec, Canada; University of South Carolina School of Medicine, United States of America

## Abstract

The ability to express exogenous cDNAs while suppressing endogenous genes via RNAi represents an extremely powerful research tool with the most efficient non-transient approach being accomplished through stable viral vector integration. Unfortunately, since traditional restriction enzyme based methods for constructing such vectors are sequence dependent, their construction is often difficult and not amenable to mass production. Here we describe a non-sequence dependent Gateway recombination cloning system for the rapid production of novel lentiviral (pLEG) and retroviral (pREG) vectors. Using this system to recombine 3 or 4 modular plasmid components it is possible to generate viral vectors expressing cDNAs with or without inhibitory RNAs (shRNAmirs). In addition, we demonstrate a method to rapidly produce and triage novel shRNAmirs for use with this system. Once strong candidate shRNAmirs have been identified they may be linked together in tandem to knockdown expression of multiple targets simultaneously or to improve the knockdown of a single target. Here we demonstrate that these recombinant vectors are able to express cDNA and effectively knockdown protein expression using both cell culture and animal model systems.

## Introduction

The past decade has seen unprecedented technological and informational advances giving today’s researcher nearly unfettered access to genome sequences and transcriptome expression analysis of both diseased and normal tissue. Traditionally, elucidating the functional role of individual genes has been reliant on genome altering technologies, these most often being transient expression systems for cultured cells and transgenesis or gene targeting technologies for *in vivo* studies. Despite their wide scale use, there are a number of drawbacks with each of these approaches. For instance, plasmid-based systems using either transfection or electroporation do not allow for efficient stable expression or knockdown of genes. Moreover, DNA uptake in particular cell types/lines can be quite poor as has been seen in primary cells. In contrast, while the manipulation of gene expression *in vivo* through mutation, over-expression, or expression ablation avoids many of the problems associated with plasmid-based systems, these techniques are often technically laborious, expensive and time consuming. Retroviral and lentiviral vectors offer the ability to efficiently transfer genetic material to multiple cell types for long-term expression both *in vitro* as well as *in vivo* and in the case of lentiviruses, allow for non-dividing cells to be transduced [1], [2]. Thus, these vectors represent an important “middle ground” between transient plasmid based systems and *in vivo* gene manipulation. To this end, there exist a number of commercial retroviral and lentiviral systems that allow cDNA overexpression or the use of RNA interference to ablate or diminish gene expression (as reviewed in [3], [4]).

The discovery of RNAi has revolutionized the manner in which endogenous gene expression can be manipulated, enabling researchers to test the consequences of loss-of-functional expression for nearly any gene. Stable knockdowns were initially achieved using RNA polymerase III promoters via H1 or U6 driven expression of short-hairpin RNAs (first demonstrated in [5], [6], reviewed in [7]). While these Pol III promoters drive high-levels of shRNA expression, their usefulness is severely limited since most are neither tissue specific nor inducible (see [8], [9] for a notable exceptions). More recently, shRNA sequences embedded in microRNA (typically human miRNA-30-based) have allowed for stable expression of shRNAs from RNA polymerase II promoters [10]. As these are Pol II initiated transcripts, they can be manipulated to permit stable, inducible, or tissue-specific expression in viral vectors (reviewed in [9], [11]). While commercially available vectors to overexpress cDNA or knockdown a single gene exist, they often constrain researchers to a handful of selectable markers (most commonly puromycin) and are available at considerable monetary expense, making their routine use impractical.

Classical restriction enzyme digestion and ligations technologies, while useful, are being superseded by ligation-independent methods [12]–[17]. These methods are less labour intensive, increase cloning efficiency and are amenable to high throughput approaches. Of these methods, Gateway cloning technology has been adopted by many due to its versatility, precision and ease of use. It is based on λ bacteriophage site-specific recombination [18] and exploits the specificity and reversible directionality of recombination reactions. In λ bacteriophage infections, the phage integrates into the bacterial genome via recombination between attP/attB sites (within the phage and bacterial genomes respectively) resulting in the formation of attL/attR sites flanking the integrated phage/recombined bacterial sequences. Gateway cloning works by harnessing this site-specific recombination along with simultaneous double genetic selection, with positive selection for one drug resistance marker and negative selection for the loss of a toxic gene flanked by recombination sites. The plasmids used in these reactions are called “Entry vectors” and “Destination vectors”. An entry plasmid contains a DNA insert flanked by att recombination sites (most frequently attL1 and attL2 sites). The Destination vector is the plasmid where the DNA insert will ultimately be cloned. It contains an attR-flanked cassette harbouring the *ccdB* gene, whose product targets bacterial DNA gyrase and is toxic to most *E.coli* strains [19], save for those with a specific gyrase mutations (e.g. DB3.1 or those containing the *ccdA* gene [20]). Generally Entry vectors are kanamycin resistant while Destination vectors are ampicillin resistant. A standard ‘LR recombination’ will exchange the contents of the Entry vectors with those of the Destination vector producing an expression vector (Figure S1). Thus, when transformed into ccdB-sensitive bacteria and selected for ampicillin resistance, only recombinant expression vectors containing the Entry vector DNA insert are capable of growing. Non-recombinants (the input Entry and Destination vectors) or the other recombination product are selected against due to the absence of the correct bacterial resistance marker, the presence of the *ccdB* gene or both. This provides extremely powerful positive/negative selection for the correct recombinant such that the DNA insert is cloned in the correct orientation and with precision to predict reading frame. A further advancement to this technology allows multiple DNA inserts contained in separate Entry vectors to be cloned in a predefined order and orientation into an expression vector via MultiSite Gateway cloning [21].

Here we describe novel lentiviral (pLEG) and retroviral (pREG) systems that permit the efficient transduction of cells with one or more cDNAs and are capable of simultaneously delivering one or more miRNA30-based shRNAs (shRNAmirs) to knockdown the expression of multiple targets in mammalian cells. To permit the rapid construction of viral vectors regardless of insert sequence, our system is compatible with MultiSite Gateway cloning technology, allowing investigators to “mix-and-match” cDNAs, markers, and shRNAmirs without the need to perform difficult and time consuming multi-step cloning. We further have developed methods to rapidly produce shRNAmirs compatible with this system and, using a luciferase-based approach, to triage these for function without the need to develop stable expressing cell lines. Here we demonstrate the effectiveness of these vectors in cultured cells using image analysis, biochemical assays and biological readouts. To demonstrate their utility *in vivo*, we used these viral vectors to simultaneously express Cre recombinase and to knockdown the expression of the tumour suppressor p53 resulting in increased proliferation of the resulting tumours.

## Materials and Methods

### Ethics Statement

Intratracheal administration of viral vectors was performed under 2,2,2 Tribromoethanol anaesthesia and all efforts were made to minimize suffering. All mouse experiments were carried out in strict accordance with the recommendations in the Canadian Council on Animal Care (CCAC) “*Guide to the Care and Use of Experimental Animals*” and under the conditions and procedures approved by the Animal Care Committee of McGill University (AUP number: 5819).

### Generation of Plasmid Vectors

#### Entry plasmids

All plasmid vectors were produced using standard cloning techniques. A more exhaustive description of the protocols used, construction history and plasmid sequence are available on request. All plasmids described herein will be made available through Addgene (www.addgene.org). AttL1-attL2 flanked genes were cloned into either pENTR-D TOPO plasmids from PCR products or into pENTR1 using standard restriction enzyme based methods. DNA containing attR2-attL3 or attR3-attL4 sites separated by a multi-cloning region was synthesized by BioBasic and used to produce two pOK1/2-derived [22], kanamycin resistant entry plasmids, *pBEG R2-L3* and *pBEG R3-L4*. The multi-cloning region separating the attX-sites contained the sequence GGGCCGGCGCGGCCGCACGCGTGCTGAGGAGACATCTAGACTTTCCCTCAGCGTCGACGATATCGGCGCGCCCCCGGG. pBEG R2-i*X-R3 containing the ‘strong’ (IRES* [23]) was produced by cloning the IRES cassette from pQXIN IRES* (a gift from Daniel Gray UCSF) into the RE3-RE4 sites of *pBEG R2-L3*. *pBEG R2-IRESX-R3*, which contains the ‘weak’ IRES, was cloned from a pQCXiX-derivative containing a puromycin resistance marker (N-acetyl-transferase gene) to create *pBEG R2-iPuro-L3*. Drug resistance genes conferring neomycin, blasticidin-S (blasticidin-S deaminase) and hygromycin-B (hygromycin phosphotransferase) were excised from pQCxix-derived plasmids and cloned between BglII/EcoRV sites of pBEG R2-iPuro-L3.

A miRNA-30 cassette was synthesized by BioBasic and cloned into the NotI/EcoRV sites of *pBEG R3-L4* to create *pBEG R3-miRNA(X)-L4*. Next an EcoRI/XhoI flanked chloramphenicol-ccdB cassette was cloned into the EcoRI/XhoI sites of the miRNA-30 cassette creating *pBEG R3-miRNA(ccdB)-L4,* which greatly simplifies the cloning of novel EcoRI/XhoI flanked shRNAs.

#### Viral destination plasmids

Synthesis of a single fragment containing tandem attR1–attR4 sites was repeatedly unsuccessful. Thus, we synthesized individual attR1 and attR4 sites, and cloned them into pOK1/2 such that they were separated by a chloramphenicol resistance marker to produce *pBEG R1-ChlorR-R4*. The chloramphenicol selection cassette was PCR amplified from a lab Gateway destination vector (*gQxiPuro,* unpublished plasmid) using the following forward (5′-CACCTCTAGACTCGAGATTAGGCACCCCAGGCTTTACAC) and reverse (5′-ATATGAATTCGTCGACCTGCAGACTGGCTGTG) primers and cloned into the XbaI site of *pOK1/2 B* [22] giving *pOK1/2 B (ChlorR)*. Next, the attR1 site from *pUC57 fragment A* was cloned into this vector using BglII/NotI giving *pBEG R1-ChlorR-R4*.

To create the three way destination vector (attR1-attR3) the attR4 site was replaced with attR3 from *pBEG R3-L4* which was cut out with NheI/NgoMIV and cloned into the SpeI/XmaI site of *pBEG R1-ChlorR-R4* creating *pBEG R1-ChlorR-R3*. Finally, the *ccdB-ChloroR* cassette from *gQxiPuro* was cloned into both the *pBEG R1-ChloroR-R3* and *pBEG R1-ChloroR-R4* vectors with NotI/SalI. Once both R1–R4 and R1–R3 Gateway cassettes existed as pBEG plasmids it was possible to produce the destination vectors *pLEG* and *pREG*. To this end, the R1–R3/R4 cassettes were excised with BglII/HpaI and cloned into *pLEXiPuro* (Open Biosystems) at BamHI/HpaI sites and with SacII/HpaI into gQxiPuro at SacII/EcoRV sites. Thus, the following four destination vectors were produced: two lentiviral vectors *pLEG(R1–R3) and pLEG(R1–R4)* and two retroviral vectors *pREG(R1–R3) and pREG(R1–R4).*

All viral destination vectors produced by this system use a self-inactivating (SIN) 3′ LTR that harbours a deletion in the U3 region, rendering the LTR transcriptionally inactive. This deletion is copied to the 5′ LTR during reverse transcription preventing further viral replication and greatly reducing the likelihood that viral insertion will activate endogenous oncogenes [24], [25].

#### Luciferase reporter plasmid

A separate destination dual luciferase reporter plasmid, pCheck2 Dest (R1–R2), was created by blunt end cloning of an attR1–attR2 destination cassette (Invitrogen) into the NotI site (blunted using Klenow) of pSiP1 [26].

*miRNA-shRNA design Plasmids.* All miRNA was produced by PCR using a ∼100 bp oligonucleotide “shRNA template” and amplified with universal primers. The 5′ universal primer (5′-CACC*CTCGAG*AAGGTATATTGCTGTTGACAGTGAG ) and 3′ universal primer (5′-CCCCTT*GAATTC* CGAGGCAGTAGGCA ) were based on those used by Hannon et al. [11]. PCRs were performed using 0.5 units Phusion polymerase, 200 nM dNTP, 400 nM of each primer, 400 nM template, 704 nM DMSO with 30 cycles (10 sec 98°C, 30 sec 60°C, 60 sec 72°C).

PCR-amplified shRNA fragments were cloned between XhoI and EcoRI sites (italicized in universal primers) of the miRNA cassette. The shRNA template oligonucleotide must have a corresponding overlap with the universal primers (underlined and in green) as shown: shRNA core template = TGCTGTTGACAGTGAGCG**A**(shRNA Sequence)**C** TGCCTACTGCCTCG (bolded nucleotides can vary but cannot complement one another, see [11], [27]). shRNA structures are based on published sequences [28] all having a constant 19-bp loop sequence (X-TAGTGAAGCCACAGATGTA-X’) flanked by 19–23 nt sequences (X and X’) homologous to target (double underlined).

Mouse p53 specific shRNAs:

HP65:TGCTGTTGACAGTGAGCG**C**CCACTACAAGTACATGTGTAATAGTGAAGCCACAGATGTATTACACATGTACTTGTAGTGG**A** TGCCTACTGCCTCGGA

HP44:TGCTGTTGACAGTGAGCG**C**GGAAATTTGTATCCCGAGTATTAGTGAAGCCACAGATGTAATACTCGGGATACAAATTTCC**T** TGCCTACTGCCTCGGA

HP18:TGCTGTTGACAGTGAGCG**A**CCAGTCTACTTCCCGCCATAATAGTGAAGCCACAGATGTATTATGGCGGGAAGTAGACTGG**C** TGCCTACTGCCTCGGA

GFP or dsRed specific shRNAs:

GFP01:TGCTGTTGACAGTGAGCG**A**GCACAAGCTGGAGTACAACTATAGTGAAGCCACAGATGTATAGTTGTACTCCAGCTTGTGC**C** TGCCTACTGCCTCGGA

dsRed01:TGCTGTTGACAGTGAGCG**C**AACGAGGACTACACCATCGTTAGTGAAGCCACAGATGTAACGATGGTGTAGTCCTCGTT**G** TGCCTACTGCCTCGGA

shLuc:TGCTGTTGACAGTGAGCG**C**CCGCCTGAAGTCTCTGATTAATAGTGAAGCCACAGATGTATTAATCAGAGACTTCAGGCGG**T** TGCCTACTGCCTCGGA

#### LR recombination reactions

Two-plasmid recombination reactions were performed using LR Clonase II in a 5 µL reaction (10 fmol Entry plasmid, 20 fmol Destination plasmid, 1 µL LR Clonase II Invitrogen cat# 11791-020). Three and four plasmid recombination reactions used LR Clonase II Plus were performed in a total 5 µL (0.5 µL each of 10 fmol/µL Plasmid A (attL1–L2), Plasmid B (attR2-L3), Plasmid C (attR3-L4), 0.5 µL of 20 fmol/µL Destination Plasmid, 0.5 µL of LR Clonase II Plus cat# 12538-120). LR Clonase II reactions were incubated for 1 hour and LR Clonase II Plus reactions were incubated for 16–24 hours prior to proteinase K treatment and transformation into chemically competent DH10B bacteria.

### Tissue Cell Culture and Transfections

#### Cell culture

HEK 293T and NIH 3T3 were cultured in DMEM (Wisent) containing +10% v/v FBS, 1% penicillin/streptomycin (Wisent) and 1% v/v 1 M HEPES solution at 37°C with 5% CO_2_. Cells were trypsinized and split 1∶10 into fresh plates at regular intervals to prevent them from reaching confluence. Mouse embryonic fibroblasts were isolated as described [29] and were cultured in DMEM containing 10% FCS, 1% penn/strep. All MEFs were cultured for a maximum of 4 passages.

#### Transfections

HEK 293T cells (5×10^6^ per 100 mm dish) were transfected using a Polyethyleneimine (P.E.I.) solution at a 2.65∶1 ratio (P.E.I. mass:DNA mass) [30]. 42 µL of P.E.I. (1 mg/mL) was added to 16 µg of plasmid DNA diluted in 600 µL OMEM and incubated 30 minutes before addition to cells in DMEM supplemented with 10% FBS. Cells were incubated at 37°C overnight for 293T cells or 6 hours for NIH 3T3 cells and then the media was replaced. For luciferase assays, 5×10^4^ HEK 293T cells were seeded in each well of a 24 well dish before transfection with PEI and 0.74 µg (total) plasmid DNA per well.

### Virus Production and Infections

Lentivirus was produced by co-transfection of pAX2 (5.2 µg), pMDG (2.8 µg) and the recombinant viral plasmid (6 µg) (14 µg DNA total, PEI and OMEM ratio as described previously) into HEK 293T cells seeded at 60% confluence in 100 mm dishes. To produce retrovirus, LNXE producer lines were transfected with 16 µg of pREG plasmid. In both cases media was removed after 48 hours, filtered through a 45 µm filter and added to the recipient cells undiluted.

### Immunoblots

Stable transduced NIH 3T3 cells were left untreated or were incubated with 0.2 µg/mL doxorubicin for 6 hours. Total protein was extracted from 1×10^6^ cells lysed at 95°C in 1X Laemmli buffer. Protein was separated by 10% SDS PAGE and immunoblotted using standard methods with primary anti-p53 antibody (1∶1000 dilution, Cell Signaling cat# 2524) or anti-tubulin (1∶8000, Sigma cat# T5168) antibody and a HRP-conjugated secondary antibody (1∶2500 GE Healthcare Life Sciences cat# NA931VS).

### Luciferase Assays

HEK 293T or NIH 3T3 cells were seeded (5×10^4^ cells per well) in a 24 well dish and were incubated overnight. DNA mixes contained either a 4∶1, 2∶1 or 1∶1 molar ratio of lentiviral plasmid expressing miRNA to luciferase reporter (always using 100 ng of reporter plasmid) were made up to 0.74 µg of total plasmid DNA by adding a third recombinant lentiviral plasmid lacking the miRNA cassette. Transfections were performed using P.E.I. as described previously. Cells were washed with 1X PBS 48 hours post-transfection and then lysed in 100 µL Passive Lysis Buffer (Promega cat# E1941) per manufacturers instructions. Firefly and Renilla luciferase contents were quantified using a Tecan 200 plate reader/injector combination running i-Control software using 5 µL of HEK 293T and 20 µL NIH 3T3 lysates to maintain signal linearity. Luciferase assay solutions were from Promega (Dual-Luciferase Reporter Assay System cat# E1910) or made as described [31], [32]. 100 µL of firefly luciferase assay solution was injected per well, shaken for 2 seconds and the luminescence measurement integrated over 10 seconds, followed in the same manner by injection of100 µL of Renilla luciferase assay solution.

### Cell Imaging

Fluorescence cell imaging was acquired using a Leica DM IL LED inverted microscope with X-cite series 120 Q UV source, QICAM Fast 1394 camera attachment (Q IMAGING) and filter sets from CHROMA: CFP: ET436/20x, ET480/40 m, T455lp, GFP: ET470/40x, ET525/50 m, T495LPXR, dsRed: ET545/30x, ET620/60 m, T570lp.

### Infection and Analysis of Mouse Lungs

Lentivirus made from recombinant plasmids expressing eGFP, Cre and Luciferase was produced and concentrated by centrifugation as described in [33]. Concentrated virus was titred by infecting 1×10^5^ HEK 293T cells per well of a 6 well dish with lentiviral dilutions made in 1X PBS at either a 1∶10 or 1∶100 dilution. To each well, 10 µL or 100 µL was added in the presence of 4 µg/mL of polybrene. The proportion of eGFP-positive cells was determined by standard flow cytometry analysis 72 hours post-infection.

Equivalent infectious units of virus (1–2×10^8^ IU) were introduced into the lungs of Braf^CA/+^ mice through direct intratracheal administration (as described in [33]) after pre-treatment with sodium caprate, which enhances infection efficiency [34]. Mice were euthanized at 8 and 16 weeks after infection and the lungs were processed for histology and Ki67 as described [35]. Slides were stained with hematoxylin and eosin (H&E) and for Ki67 before being scanned using an Aperio Scanscope AT. Individual slides were analyzed using Aperio ImageScope software, in which each tumour was circumscribed to obtain the section area (µm^2^) and the percentage of Ki67-positive cells was obtained using the IHC Nuclear Algorithm.

## Results

### Development of Retroviral and Lentiviral Expression Vectors with Multiple Markers

**Figure 1:**
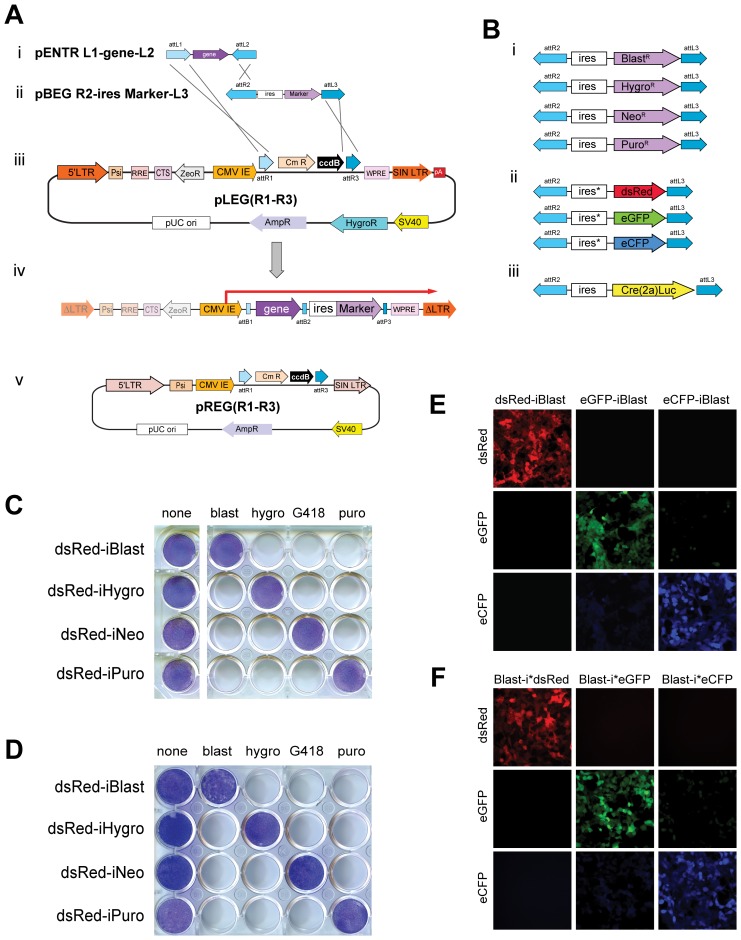
Modular design and function of pLEG/pREG viral vector expression system. A) A generalized three-plasmid LR recombination reaction depicting the insertion of a gene and selection marker into a lentiviral backbone. Each attLx site recombines with a corresponding attRx site and the order and orientation of these sites directs the formation of the recombinant attXx site as well as the insert order/orientation. AttL1-attL2 (i) and attR2-attL3 (ii) flanked entry vectors recombine with a lentiviral destination vector, pLEG(R1–R3) (iii) producing a recombinant lentiviral expression vector that when integrated contains a single CMV-driven bicistronic transcript (iv). Retroviral destination vectors (pREG) are also possible and function in the same manner (v). LTR: Long Terminal Repeat, Psi: packaging signal, RRE: Rev Response Element, CTS: central PolyPurine Tract, CMV IE: cytomegalovirus-immediate early, WPRE: Woodchuck hepatitis Post-transcriptional Regulatory Element, ΔLTR: Self Inactivated LTR. B) Drug resistance markers (i) for use with the pLEG/pREG system along with fluorophore markers (ii) and Cre2ALuc (iii) which may be inserted and expressed downstream of any attL1-attL2 flanked gene. C) Stable NIH 3T3 cell lines expressing each of the four drug resistant markers after infection by a recombinant lentiviral (pLEG) vector produced by three-plasmid recombination reaction. Giemsa staining highlights the drug resistant populations for each case. D) Stable NIH 3T3 cell lines expressing each of the four drug resistant markers after infection by a recombinant retroviral (pREG) vector – as in (C). E) Stable HEK 293T cell lines expressing each of the three upstream fluorophore markers after infection by a recombinant lentiviral (pLEG) vector produced by three-plasmid recombination reaction. F) Stable HEK 293T cell lines expressing each of the three downstream fluorophore markers – as in (E). Psi: RNA packaging symbol; SIN LTR: self-inactivating long terminal repeat; WPRE: Woodchuck hepatitis virus post-transcriptional element; CmR/ccdB: Chloramphenicol resistance/ccdB cell death cassette; ZeoR: Zeocin resistance cassette; pA: poly adenylation signal; AmpR: Ampicillin resistance gene; HygroR: Hygromycin resistance gene; pUC ori: pUC origin of replication; RRE: HIV rev response element; ΔLTR: integrated transcriptionally inactive LTR. BlastR: blasticidin resistance gene; NeoR: Neomycin resistance gene; PuroR: Puromycin resistance gene; ires: internal ribosomal entry sequence; ires*: modified internal ribosomal entry sequence with enhanced activity; dsRed: Discosoma red fluorescent protein; eGFP: Enhanced green fluorescent protein; eCFP: Enhanced cyan fluorescent protein; Cre(2a)Luc: Cre recombinase T2A fusion to firefly luciferase for polycistronic expression. blast: Blasticidin; hygro: Hygromycin; G418: Geneticin; puro: Puromycin.

Retroviral and lentiviral vectors are efficient vehicles to stably introduce genetic material to a wide variety of cell types, both in cell culture and in whole animals (reviewed in [36]). To facilitate the process of generating such viruses we sought to create lentiviral expression vectors capable of expressing a cDNA and marker (drug resistance, fluorophore, etc.) from bicistronic mRNA by modifying an existing commercial lentiviral vector, pLEX (OpenBiosystems). This self-inactivating [24], [25] lentiviral expression vector was altered to contain a ccdB cassette flanked by 5′ attR1 and 3′ attR3 sites placed downstream of CMV promoter/enhancer sequences creating a Gateway-compatible Destination vector called pLEG(R1–R3) (Figure 1Aiii). This vector was designed for use in three-plasmid recombination reactions with Entry vectors containing a cDNA between attL1-attL2 sites (Figure 1Ai) and genetic markers between attR2-attL3 sites (Figure 1Aii). Following recombination the ccdB cassette is replaced with desired Entry sequences. Integrated viruses express a single bicistronic transcript emanating from the CMV promoter/enhancer (Figure 1Aiv). In a similar fashion a SIN-retroviral vector (pQCxix, Clontech) was altered to create the Destination vector pREG(R1–R3) (Figure 1Av).

#### Primary expression of cDNA

cDNAs are cloned between attL1–attL2 sites (Figure 1Ai) to create a kanamycin resistant “Entry vector”. New cDNAs may be cloned into these vectors directionally via traditional restriction enzyme based means, captured from a PCR product using efficient TOPO systems (pENTR-D TOPO) or by performing a BP recombination reaction (Invitrogen). Alternatively, human, mouse and rat genes are available commercially as cDNAs or ORFs in attL1–attL2 Entry vectors and are fully compatible with our system (e.g. DNASU plasmid repository, GeneCopoeia). Moreover, a number of attL1–attL2 Entry vectors exist that contain different tags to allow for detection (antibody epitopes), purification (TAP or GST tags), and to induce dimerization [37].

#### Selection cassettes

We created a series of plasmids encoding either drug resistance or fluorescent protein markers downstream of an internal ribosomal entry sequence (IRES) between attR2–attL3 sites (Figure 1B and Figure S2A). This construction allows for bicistronic expression of the marker along with an upstream cDNA (between attL1–attL2). Specifically, these selection markers confer resistance to blasticidin, hygromycin, puromycin, and neomycin (Figure 1Bi) or allow for the expression of eGFP, eCFP, and dsRed (Figure 1Bii). For *in vivo* experiments we made use of *Thosea asigna* virus-derived 2A peptide [38], [39] to express both Cre recombinase and firefly luciferase (Figure 1Biii). The 2A peptide allows for expression of two distinct proteins encoded in a single open reading frame when separated by this highly conserved 18–22 amino acid sequence through a process of translational skipping or cleavage [40], [41]. Cre-2a-Luciferase allows for the Cre mediated recombination of appropriately modified target genes while simultaneously tracking infection using luciferase activity as a surrogate. Here we use two distinct IRES sequences differing only at the 3′ end and subsequently referred to as either ‘strong’ or ‘weak’, reflecting their efficacy at expressing the downstream cDNA [23]. The ‘weak’ IRES (3′ sequence: GATGAT**AAGCTT**GCC) was used for all drug selection markers while the ‘strong’ IRES (3′ sequence: GATGAT**AATATG**GCC) was used for the fluorophores in order to achieve the higher levels of expression necessary for their visualization.

Recombinant lentiviral and retroviral vectors were produced containing dsRed upstream (visualized to verify expression but not shown) and each of the four drug resistance genes downstream (e.g. pLEG dsRed-iPuro as in Figure 1Aiv). Lentivirus and retrovirus produced from these vectors were transduced into NIH 3T3 cells. Two days post-infection, stably expressing cells were selected with blasticidin, hygromycin, neomycin (G418) or puromycin to determine functionality and specificity of these markers. In each case, lentiviral (Figure 1C) and retroviral (Figure 1D) vectors expressed the upstream cDNA (here dsRed, others not shown) and conferred resistance to the appropriate drug. The proper functioning of the fluorescent protein markers (eGFP, eCFP, dsRed) either up or downstream (after a ‘strong’ IRES) was tested with recombinant lentiviral vectors. Each of these constructs encoded a blasticidin resistance gene and was transduced into HEK 293T cells. Drug-selected cells were visualized by fluorescence microscopy (Figure 1E, F). Using the ‘strong’ IRES we demonstrate comparable levels of fluorescent protein expression when placed upstream or downstream of the IRES. Together this data demonstrate that these vectors can be used to efficiently deliver cDNAs to cells using a number of drug selectable markers as well as identifying infected cells with fluorescent proteins.

#### miRNA cassettes

**Figure 2:**
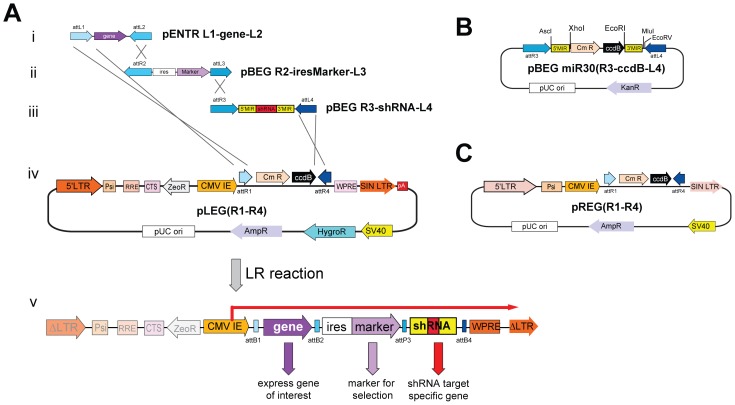
Overview of pLEG/pREG vectors to express shRNAmirs. A) A typical four-plasmid LR recombination reaction showing the insertion of a gene (i), selection marker (ii) and miRNA cassette (iii) into pLEG(R1–R4) (iv) to produce a recombinant lentiviral virus (v). B) Schematic of the miRNA cassette and entry plasmid showing the Chloramphenicol resistance/ccdB cell death cassette situated between XhoI/EcoRI sites of pBEG miRNA(R3-ccdB-L4) to increase the cloning efficiency of novel shRNAs. C) The retroviral destination vector pREG(R1–R4) used in four-plasmid LR recombination reactions – functions as in (A). KanR: Kanamycin resistance gene; 5′MIR: 5′miR30 sequences; Cmr: chloramphenicol resistance marker; 3′MIR: 3′miR30 sequences.

To increase the utility of these vectors we sought to enable simultaneous cDNA expression and ablation of target gene expression. To achieve this goal we created a Destination vector called pLEG(R1–R4), which contains attR1 and attR4 sites to allow for a four plasmid LR recombination (Figure 2A). Based on the work of Hannon and colleagues [10], [27], we generated an Entry vector encoding a miR30-embedded shRNA to knockdown targeted gene expression. Specifically, shRNAs are cloned into a modified miRNA-30 between unique XhoI and EcoRI sites such that the shRNA-miR30 (herein called shRNAmir) is flanked by attR3-attL4 sites (Figure 2B and Figure S2B) allowing their placement downstream of the cDNA/selection cassette after recombination. It should be noted that the presence of an upstream cDNA enhances knockdown in miRNA-shRNA based vectors [10]. To make the cloning of novel miRNAs more facile we created the entry vector: pBEG R3-miRNA(ccdB)-L4. This plasmid contains the *ccdB* gene, whose expression is toxic to most bacteria, cloned between the XhoI and EcoRI sites, embedding it in miR-30. shRNAmirs are cloned into the miRNA-30 cassette directly from a universal PCR reaction by first treating the PCR product with proteinase K to inactivate the polymerase and then heat inactivating the proteinase K before digesting with XhoI/EcoRI and ligating it into the miRNA-30 cassette (Figure S2D). A successfully cloned shRNA replaces the *ccdB* negative selection gene thereby dramatically increasing shRNAmir cloning efficiency. Using this approach we routinely need only pick a single colony to obtain the correct clone. Thus, new shRNAmirs may be used immediately in recombination reactions to produce viral expression plasmids for triage and testing. Additionally, when using this entry vector shRNAmirs may be cloned in series *in situ* by cutting out a given miRNA-30 cassette using AfeI/MluI and cloning it into a recipient entry vector between PmeI/MluI behind another miRNA-30 cassette (Figure S2E-H).

### Testing the Activity of shRNAmirs

#### Tandem shRNAmirs can target different genes

**Figure 3:**
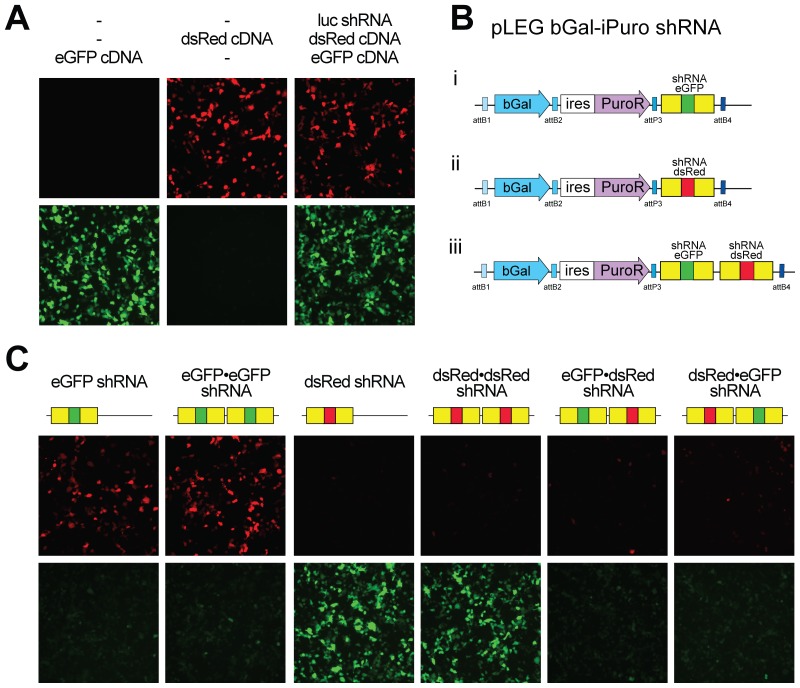
Efficient knockdown of one or more genes using a pLEG. A) Transfection of HEK 293T cells with recombinant lentiviral vectors expressing either eGFP or dsRed with or without a recombinant lentiviral vector expressing miRNA to firefly luciferase as indicated. Cells were visualized 48 hours post transfection for red and green fluorescence. B) A graphic showing the general structure of the recombinant lentiviral vectors used in this experiment with single miRNA cassettes targeting eGFP (i), dsRed (ii) and both (iii) miRNAs daisy-chained together. C) Cotransfections of recombinant lentivirus containing fluorophore miRNA cassettes (single and daisy chained) as well as both eGFP and dsRed (pLEG fluorophore-iBlast) into HEK 293T cells. Cells were visualized 48 hours post transfection for eGFP and dsRed expression. bGal: Beta-Galactosidase.

To test the function of this shRNAmir construction we chose to initially target the expression of fluorescent proteins. shRNAs were designed to target dsRed, eGFP and firefly luciferase (as a control). Long oligonucleotides (∼100 nt) served as templates for PCR and the products were cloned into pBEG R3-miRNA(ccdB)-L4 as described (Materials and Methods, depicted in Figure S2D, E). These shRNAmirs were then recombined into a lentiviral destination vector along with β-Galactosidase, cDNA and a puromycin drug resistance marker. The resultant shRNAmir-containing lentiviral plasmids were co-transfected along with vectors expressing dsRed and eGFP into HEK 293T cells. After 48 hours the cells were visualized for eGFP and dsRed expression as a read out of miRNA activity. As expected the shRNA targeting luciferase, while effective at reducing luciferase activity (Figure S3C) had no effect on eGFP or dsRed expression (Figure 3A) whereas an eGFP-shRNA encoding vector (Figure 3Bi) specifically reduced eGFP but not dsRed expression (Figure 3C). Similarly, expression of the dsRed shRNA (Figure 3Bii) extinguished expression of dsRed but not eGFP. These cells efficiently expressed the encoded β-Galactosidase yet there remained a few cells where fluorescent protein expression could be found. We speculated that because of the relatively long half lives of eGFP and dsRed, being approximately 26 hrs [42] and 4.2 days [43] respectively, this residual expression may be a reflection of protein stability. To further reduce fluorescent protein expression we encoded two identical shRNAmirs in tandem (e.g. eGFP•eGFP shRNA). Here we detected no obvious benefit of using multiple identical miRNAs to the eGFP or dsRed in our tests, however targeting other genes we have detected an added benefit to reiterating shRNAmirs (Figure 3C and data not shown). Similar results were obtained when analyzed by flow cytometry (Figure S3A).

To determine whether tandem shRNAmirs could be used to simultaneously knockdown expression of two or more genes, lentiviral vectors encoding tandem shRNAmirs to dsRed and eGFP (e.g. Figure 3Biii) were transfected along with eGFP and dsRed expression vectors. These ‘daisy chained’ shRNAmirs efficiently extinguished expression of both genes (Figure 3C). Thus we have shown that ‘daisy chaining’ shRNAmirs in this way allows for the knockdown of multiple targets. This may be advantageous in situations where it is desirable to target multiple members of a gene family or genes encoding different arms of a transduction pathway.

#### Activity of shRNAmir to endogenous gene

**Figure 4:**
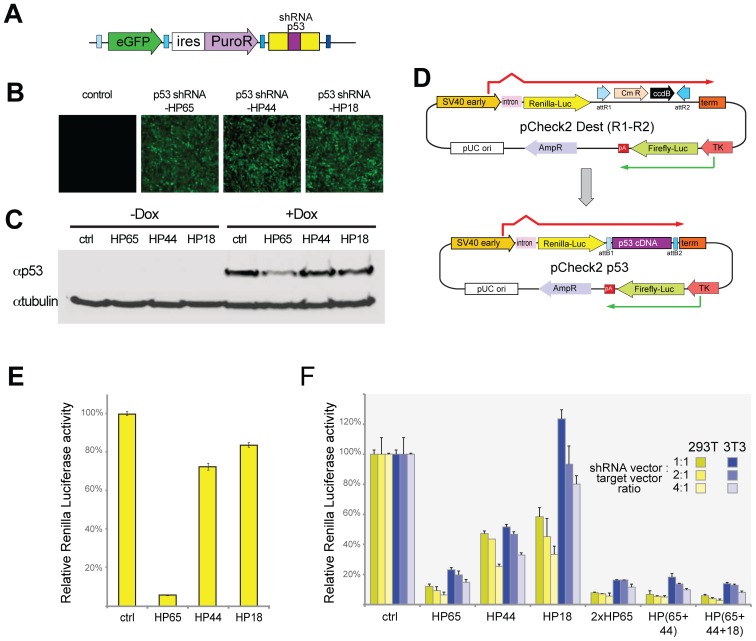
Rapid screening of p53 knockdown using stable and transient pLEG shRNAmir expression. A) A schematic depicting the general structure of the pLEG lentiviral expression vector after recombination with an shRNAmir cassette targeting p53. B) Stable cell populations were generated by infecting NIH 3T3 cells with lentivirus and selected for puromycin resistance. Each stable population expresses a unique miRNA cassette to p53 (HP65; HP44; HP18). Levels of expression are indicated by eGFP. C) A Western showing lysates from the stable cell lines (B) as well as the untransfected cells with and without doxorubicin induction. D) An overview of the pCheck2 system for rapidly triaging novel miRNAs before and after recombination to insert p53 cDNA downstream of Renilla luciferase. The recombination reaction is performed between attL1–attL2 and attR1–attR2 sites allowing for compatibility with all standard cDNA entry plasmids (attL1–attL2). E) Transfections of the pCheck2 p53 dual luciferase reporter plasmid into stable cell populations (from C) expressing the three miRNAs to p53 as well as uninfected control cells. The relative activity of Renilla luciferase is displayed as a percent ratio of firefly to Renilla activity scaled to the control cells (miRNA to dsRed – dsRed01). F) Transfections of the pCheck2 p53 along with pLEG vectors containing control shRNAmir (to dsRed) or to p53 (single and daisy chained cassettes) were performed with three different ratios of miRNA to pCheck2 target (1∶1, 2∶1, 4∶1) in both NIH 3T3 and HEK 293T cell lines. Luciferase activity was measured as in (E) and is displayed as a relative percent scaled to the control transfections. SV40 early: SV40 virus promoter/enhancer; TK: thymidine kinase promoter; pA: poly adenylation signal.

Having demonstrated the effectiveness of these vectors against transfected targets we sought to demonstrate their efficacy against an endogenously expressed gene. To this end, we first generated three shRNAmirs to mouse p53. These sequences were acquired either from a commercial source (HS18, Open Biosystems) or based on previously published sequence (HP65, [44]) and from RNAi codex (HP44). HP65 and HP44 sequences were adapted to work with our universal primer system for amplifying shRNAmirs by extending them at the 5′ and 3′ ends with corresponding homology to miRNA-30 (see Materials and Methods). These p53 shRNAmirs were cloned into attR3-attL4 entry vectors and then recombined into an attR1–attR4 lentiviral destination plasmid along with eGFP cDNA and a puromycin drug resistance marker (Figure 4A). Lentiviruses were produced, used to infect NIH 3T3 cells and pooled puromycin-resistant clones were obtained for each construct (Figure 4B). p53 levels are characteristically low in non-transformed cells, in part due to degradation mediated by Mdm2 (Hdm2 in human cells), which physically associates with p53 [45]. DNA damage activates ATM/ATR kinases, which phosphorylate Mdm2 ultimately freeing p53 from negative regulation and leading to elevated p53 levels [46]. Thus we treated cells with doxorubicin as a method of elevating p53 levels [47]. Cells were left untreated or were treated with doxorubicin for 6 hours to induce p53 expression. Of the shRNAmirs tested, only HP65 was able to consistently reduce p53 expression (Figure 4C).

Given that p53 protein is subject to Mdm2 mediated degradation and that p53 induces Mdm2 transcription [48], we further tested the effectiveness of these p53-shRNAmirs to target p53 mRNA using a readily quantifiable readout that is independent of p53 protein stability. Here we employed the psiCHECK-2 plasmid system (Promega). This system is based on the observation that efficient translation initiation requires the formation of a lariat structure between the 5′-cap and the polyadenylation-tail of mRNAs [49], [50]. shRNA targets are cloned downstream of Renilla luciferase but upstream of a polyadenylation sequence such that the target is contained within the same transcript but is preceded by a stop codon [51]–[53]. Cleavage of mRNA at an shRNA target site will prevent the efficient translation of Renilla luciferase encoded upstream. psiCHECK-2 additionally contains an independent transcriptional unit encoding Firefly luciferase to serve as an internal transfection efficiency control. We generated a Gateway compatible destination vector, pCheck2 Dest (R1–R2) (Figure 4D) into which we cloned mouse p53 cDNA (to create pCheck2 p53) to serve as an shRNA target.

The presence of firefly luciferase in the psiCHECK-2 derived vectors allows normalization of the Renilla luciferase expression that monitors the RNAi effect. pCheck2 p53 was transfected into NIH 3T3 cells that had been either mock infected or stably transduced with lentiviruses encoding shRNAs targeting p53 (HP65, HP44, HP18). The relative amounts of Renilla vs. firefly luciferase were then quantified (Figure 4E). Again cells expressing HP65 displayed effective knockdown whereas the HP44 and HP18 displayed only moderate knockdown. This demonstrated that the psiCHECK-2 system can be used an effective readout for expression knockdown.

#### A method to rapidly determine effectiveness shRNAmir

This approach required that we make stable cell populations expressing each lentiviral vector prior to testing the effectiveness of the shRNAmir against its target. We tested the possibility of screening shRNAmir knockdown using transient transfection of psiCHECK-2 derived plasmids into HEK 293T and NIH 3T3, the latter to directly compare to the stable expressors. NIH 3T3 or HEK 293T cells were transfected with the same lentiviral plasmid vectors along with pCheck2 p53 at different shRNA vector to target ratios and assessed for relative Renilla luciferase expression. In both cell lines HP65 efficiently decreased expression in a p53-target-dependent fashion (assessed using different cDNAs in pCheck2 to test specificity, not shown). We did detect a difference in the effectiveness of knockdown between the cell lines for HP18, with ablation most effective in HEK 293T cells. Given that these lentiviral vectors each contain the SV40 origin and that HEK 293T cells contain large T [54], we hypothesize that the difference is due to the replication of these vectors in HEK 293Ts [55] thus leading to increased amounts of the shRNA relative to those in NIH 3T3s.

To determine whether we could obtain a further reduction in p53 expression we generated tandem shRNAmirs containing 2 or 3 shRNAmirs with either the same (2xHP65) or different shRNAmirs (e.g. HP(65+44+18)). In these instances we found a slight increased knockdown with additional shRNAs (Figure 4F). These results demonstrate that one can screen candidate shRNAs using transient transfection of psiCHECK-2 derived vectors into the cell of choice in order to triage potential shRNAmir on the basis of effectiveness. This procedure can be streamlined such that the time from obtaining the shRNAmir template (the long oligonucleotide) to assessing knockdown efficiency is less than 8 days.

### Biological Activity of pLEG Vectors

Primary cells proliferate for a finite number of cell divisions before entering an irreversible growth arrest termed replicative senescence (first described by Hayflick and Moorhead [56], reviewed in [57]). These cells senesce due to telomere attrition [58], [59] and while oncogenic Ras readily transforms immortalized cells, it fails to transform primary cells due to the induction of senescence [60]. This failure in oncogenic transformation is not due to the telomere erosion [61] but is thought to reflect the engagement of a built-in tumour suppressor mechanism. Indeed, the loss of specific tumour suppressor genes has been shown to render primary cells permissive to oncogenic transformation. This is particularly evident in primary mouse embryonic fibroblasts (MEFs) where p53 loss is sufficient to permit immortalization and Ras induced transformation [60]. Having demonstrated the effectiveness of these lentiviral vectors expressing shRNAmirs to extinguish expression of targeted genes we sought to demonstrate biological function of shRNAmir-directed gene inhibition.

**Figure 5:**
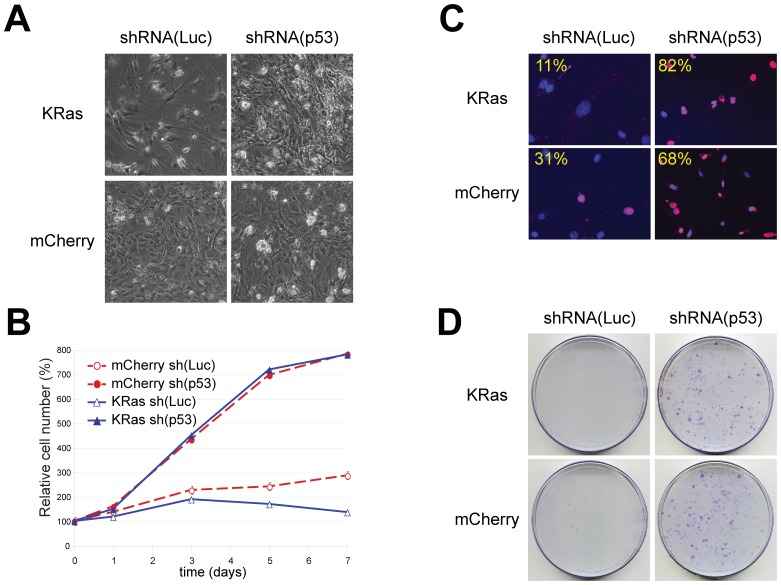
Functional knockdown of p53 in MEFs. MEFs were infected with lentiviruses expressing shRNAmirs targeting firefly luciferase (shRNA(Luc)) or p53 (shRNA(p53)) as indicated. Cells were subsequently transduced with pLEG vectors encoding KRas^V12^ (pLEG KRas^V12^-iBlast) or mCherry (pLEG mCherry-iBlast) where indicated. A) Characteristic cell morphology 14 days post-infection. Photographs are at the same magnification. Note the flattened morphology and sparse number of shRNAmir(Luc) cells expressing Kras^V12^ (top left). B) Representative growth curves corresponding to MEFs transduced with a control shRNAmir vector (open symbols) or with shRNAmir targeting p53 vector (closed symbols) and with expression vectors encoding mCherry (dashed red lines) or Kras^V12^ (solid blue lines). Each curve was performed at three times using MEFs obtained from independent embryos and each time point was determined in triplicate. C) Cell proliferation as measured by the percentage of positive cells after a 24 hr pulse with BrdU. Overlayed images of DAPI stained nuclei and BrdU-positive cells are pseudo-coloured Red. Percentages of BrdU positive nuclei were obtained by counting at least 100 nuclei from random fields. D) MEF cells transduced with and selected for the indicated viruses were plated at low density 5000 cells/100 mm dish. Plates were fixed and stained with crystal violet after 10 days of growth. Viruses used shRNA(Luc): pLEG eGFP-iPuro shRNA(luc); shRNA(p53): pLEG eGFP-iPuro shRNA(HP65); mCherry: pLEG mCherry-iBlast; KRas: pLEG KRas^V12^-iBlast.

MEFs were transduced with lentiviruses encoding a fluorescent protein along with a selectable marker (eGFP-iPuro) and either an shRNA to firefly luciferase as a control or HP65 to target p53. Following drug selection these cells were infected with viruses encoding a blasticidin resistance marker and either a fluorescent protein mCherry or oncogenic KRas^V12^. Cells were rapidly selected with blasticidin. While the mCherry containing cells that expressed either a control shRNA or HP65 were morphologically indistinguishable, the KRas^V12^ cells were different. Specifically the KRas^V12^ cells expressing the control shRNA were larger and flatter than either mCherry expressing cells and appeared to be growth arrested. KRas^V12^ cells harbouring the p53-shRNAmir grew to a higher cell density and displayed a morphology distinct from KRas^V12^ -control shRNAmir or cells expressing mCherry (Figure 5A).

The proliferative properties of these cell populations were assessed with growth curves, colony formation assays and by BrdU incorporation. Cells transduced with the control luciferase shRNAmir along with mCherry cDNA increase in number steadily over 7 days (Figure 5B) and eventually formed small colonies when plated at low densities (Figure 5D). At 8 days, 31% of the mCherry control cells were found to incorporate BrdU over a 24 hour pulse (Figure 5C). In contrast, control shRNAmir expressing cells transduced with KRas^V12^ cDNA failed to increase in number, did not form colonies when plated at low densities and had a much reduced BrdU incorporation rate (11%). These data are consistent with those observed by others, that oncogenic Ras induces growth arrest in primary cells [6], [29], [60], [62], [63]. Transduction with shRNAmirs targeting p53 lead to increased proliferation and efficient colony formation for both mCherry and KRas^V12^ expressing cells. Moreover, unlike the growth arrest induced by KRas^V12^ expression in control luciferase shRNAmir cells, KRas^V12^ expression coupled with p53 targeting lead to a large increase in the number of BrdU positive cells (>80%). Together these data demonstrate that pLEG vectors can functionally deliver cDNAs as well as knockdown of endogenous gene expression.

### *In vivo* Transduction of pLEG Lentiviral Vectors

**Figure 6:**
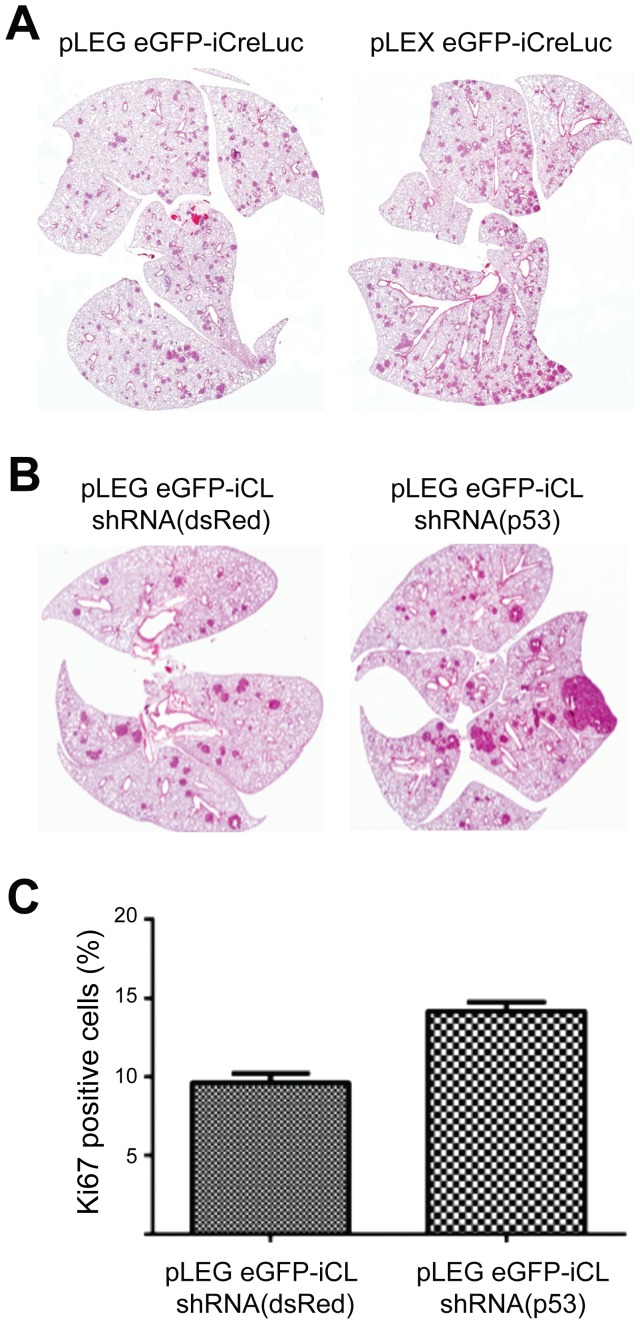
Induction of Lung Tumours Using pLEG Lentiviral Vectors. BRaf^CA/+^ mice were intratracheally infected with 1–2×10^8^ IU of the indicated purified lentiviruses and were analyzed at 8 A) and 16 (B, C) weeks post infection. Representative hematoxylin and eosin staining of histological sections of lung sections are depicted (A, B). C) Quantification of proportion of Ki67 positive nuclei within adenomas. (p<0.01, 2-sided t-test).

The direct modification of the mouse genome remains a technically challenging, costly and time-consuming endeavour. With this in mind we sought to determine if our vectors would function to transduced cells *in vivo*, in a living animal. Here we chose to infect mice carrying a Cre-conditionally active BRaf allele, *BRaf^CA^* [35]. *BRaf^CA^* mice express wild-type BRaf prior to Cre-mediated recombination after which oncogenic BRaf^V600E^ is expressed at physiological levels. We have previously shown that lung specific BRaf^V600E^ expression leads to the formation of lung adenomas within as little as 8 weeks post-BRaf^V600E^ induction using adenoviral Cre vectors. Lentiviral vectors were constructed to express eGFP, which can be used to determine viral titre, along with the Cre-2a-Luciferase fusion. Two lentiviral vectors were constructed (Figure S4): pLEX eGFP-iCreLuc, which was created using classical restriction enzyme based cloning and, pLEG eGFP-iCreLuc, which was created via recombination based methods. Lentiviruses were introduced to the lungs of *BRaf^CA/+^* mice intratracheally using published methods [33]. Mice were monitored and were subsequently analyzed for signs of lung tumours (Figure 6A). Both lentiviral vectors were capable of forming a large number of tumours in infected BRaf^CA/+^ mice but not in control wild-type mice. Additionally, lentiviral vectors lacking Cre recombinase fail to form tumors in BRaf^CA/+^ mice (Figure S5 and not shown). This data indicated that these lentiviral vectors are able to infect lung epithelial cells, integrate, and express their encoded cDNAs. We did not detect any difference between these constructions *in vivo* (Figure 6A) or in cultured cells (not shown), suggesting that the use of this Gateway recombination cloning approach does not impact the effectiveness of such vectors.

BRaf^V600E^ expression in the lung results in the formation of benign lesions that, with time, eventually cease proliferating due to the engagement of growth inhibitory signals that are mediated in part through p53 [35]. To determine if shRNAmirs can be used *in vivo* to inhibit the functional expression of genes we targeted p53 while simultaneously inducing BRaf^V600E^ through the expression of Cre recombinase (here as Cre-2a-Luciferase). To this end pLEG(R1–R4) was used to rapidly create lentiviral vectors encoding Cre recombinase in addition to either an shRNAmir targeting p53 (HP65) or a control shRNA, targeting dsRed (see Figure S3D and S3E). Purified and concentrated viruses were transduced into the lungs of BRaf^CA/+^ mice. The lungs were analyzed histologically (Figure 6B) and for signs of proliferation. We found that HP65 expression lead to an increase in proliferation as measured using Ki67 expression (Figure 6C). These data demonstrate that these lentiviral vectors can be used to alter cells *in vivo* to express cDNAs as well as to functionally ablate the expression of targeted genes.

## Discussion

There are a number of methods to manipulate gene expression. These systems run the gamut from: transient expression systems using protein transduction [64], direct RNA transfection [65], [66], plasmid-based expression vectors, or adenoviral vectors [67]; to more stable non-genomic systems using RNA based Sendai viral systems [68], episomally maintained plasmids [69], [70], or AAV [71], [72]; to integrated transposons [73], [74], or retroviral and lentiviral vectors. Here we have designed both retroviral and lentiviral vectors to create viruses that are capable of simultaneously expressing two or more genes while extinguishing the expression of at least two different endogenous genes in a single viral entity using Gateway technology.

A number of lentiviral and retroviral systems exist that permit the expression of cDNA and/or shRNAmirs [8], [10], [37], [75]–[81]. These previous efforts have proven to be very useful, yet among them none exist that combine the modularity of the pLEG/pREG system with the restriction enzyme independent cloning to allow the user to alter the desired cDNAs, markers and shRNA simultaneously. Since this recombination-based cloning method is extremely efficient (typically in the upper 90% range when input DNA concentrations are adjusted as detailed in Materials and Methods), and with the use of specific bacteria (DH10B) where white/clear colony screening is possible [82], one need only pick a single bacterial colony permitting the cloning of many plasmids in parallel. Thus, the pLEG/pREG system permits the production of these vectors with high efficiency for medium/high-throughput vector construction.

The strength of our system lies in its flexibility: there are 4 types of viral vectors, two lentiviral and two retroviral each allowing either a 3-way or 4-way recombination; cDNAs are cloned in standard attL1-attL2 flanked Entry plasmids; markers exist downstream of an IRES element in attR2–atL3 flanked Entry vectors; and shRNAmirs are encoded in plasmids flanked by attR3 and attL4 sites. Any vector can be made by choosing an expression vector, a cDNA, a selective marker, and, if desired, an shRNAmir plasmid. This modularity will permit labs to develop and share their own specific banks of Entry vectors. cDNAs can be obtained from commercial and open sources [83], by PCR mediated cloning into Entry vectors or by standard cloning techniques. Furthermore, ORFs can be fused to a number of N-terminal or C-terminal tags for protein purification or immuno-detection [37]. The repertoire of markers in attR2–attL3 flanked plasmids can be expanded to include additional fluorescent proteins [84] and cell surface markers for FACS sorting (e.g. IL3R and NGFR [10], [85]) or additional genetic markers. Individual cDNAs can be combined with different markers in pREG/pLEG vectors to introduce genes sequentially into cells for biochemical, image or functional analysis.

To facilitate the rapid identification of functional shRNAmirs we developed a method to rapidly produce (by PCR) and triage (by dual-luciferase assay) novel shRNAmirs for use with this system. When this method is used there is no need to purify the PCR product before cloning into the recipient miRNA-30 cassette and a ccdB negative selection cassette ensures that nearly every colony after selection will be correct. This facilitates the routine synthesis and cloning of large numbers of shRNAmirs into attR3–attL4 entry vectors. Lastly, we developed a luciferase-based assay as a surrogate for directed mRNA degradation. Others have used this approach to demonstrate the activity and specificity of novel miRNAs for specific mRNAs [26], [86]. The use of pCheck2 Dest (R1–R2) has allowed us to quickly determine if a given shRNA can be used to efficiently knockdown expression of a target without the development of stable shRNAmir expressing cell lines. Indeed, this has allowed us to rapidly determine knockdown mediated by a large number of different shRNAmirs to >10 different genes and this has correlated with protein knockdown (Figure S3D, de Bruyns. et al, in preparation). Moreover the cloning and triage of novel shRNAmirs can be streamlined such that the time from obtaining the shRNAmir template (the long oligonucleotide) to assessing knockdown efficiency is less than 8 days. Thus, a library of effective shRNAmirs can readily be developed for a number of targets simultaneously.

We have used these viral vectors to infect cells in culture as well as mouse lung cells *in vivo*. These viruses can be engineered to express multiple cDNAs. Thus they can be used to determine whether a given gene has oncogenic potential alone, or in combination with other genetic perturbations. The additional ability to ablate gene expression enables researchers to investigate the role of specific genes. This is most well suited to investigating tumour suppressor gene function, without the need to generate conditional null alleles. Thus, this Gateway compatible viral construction system represents an important addition to the modern biochemical toolbox giving researchers the ability to routinely produce novel viral expression vectors using essentially any combination of cDNA and miRNA-30 for a multitude of purposes to study gene function *in vitro* and *in vivo*.

## Supporting Information

Figure S1 **Standard and multi-plasmid Gateway recombination reactions.** A) A typical “Entry vector” and “Destination vector” (i). The entry vector contains a gene insert flanked by attL1 and attL2 sites and a kanamycin resistance marker. A hypothetical Destination vector (ii) is depicted with a number of specific vector elements (non-labelled arrows and rectangles), a promoter placed upstream of a Gateway cassette with attR1 and attR2 sites flanking both chloramphenicol resistance and ccdB genes and an ampicillin resistance marker. The products of an LR reaction (iii) are an Expression vector containing all the elements of the destination vector outside the attR1 and attR2 selection cassette with the gene now flanked by attB1 and attB2 sites and a “Donor vector” (iv) containing the ccdB/chloramphenicol genes flanked by attP1/2 sites. Of the four plasmids (Entry, Destination, Donor and Expression vectors) only the Expression vector has an ampicillin resistance marker and lacks the Gateway selection cassette. B) A multi-plasmid Gateway recombination is depicted with three “Entry vectors” each conferring kanamycin resistance and having distinct DNA1, DNA2 and DNA3 flanked respectively by attL1-attL2, attR2-attL3, and attR3-attL4. These Entry vectors are selected against with ampicillin. A hypothetical Destination vector, DEST (R1–R4), compatible with a 4-way recombination is depicted. DEST (R1–R4) confers ampicillin resistance and contains the CmR/ccdB cassette. The Gateway cassette in the Destination vector is selected against in most bacteria. Following a multi-plasmid LR reaction, the reaction is transformed into ccdB-sensitive bacteria and plated on ampicillin containing plates, which selects for the Expression vector. Note: DNA1, DNA2, and DNA3 are now flanked with attB sites and are in a specific order dictated by the original sites flanking each. CmR/ccdB: Chloramphenicol resistance/ccdB cell death cassette; pUC ori: pUC origin of replication; AmpR: Ampicillin resistance gene; KanR: Kanamycin resistance gene; All Gateway recombination sites are in blue.
(TIF)

Figure S2 **Additional Entry vectors.** A) Prototypical ires-gene containing entry vector for use with 2 and 3 way recombination reactions showing a strong ires sequence (ires*) flanked by unique restriction sites for cloning up and downstream as well as attR2 and attL3 sites to allow for recombination behind an attL1-attL2 containing entry vector. B) A prototypic Entry vector containing a human miRNA-30 cassette with the shRNA sequence is cloned between unique XhoI and EcoRI sites. This cassette is flanked by a number of unique restriction enzyme sites that allow for cloning up and downstream of the cassette as well as attR3 and attL4 gateway sites to allow recombination directly after the ires-marker containing entry vector (A). C) pBEG miR30(R3-ccdB-L4) was modified to place a chloramphenicol resistance gene along with ccdB between the XhoI and EcoRI sites providing negative selection when cloning in new shRNA sequence. D) General method for the PCR amplification of novel shRNAs from a ∼100 bp oligonucleotide core (e.g. shRNA2) with two universal primers (red arrows). After high fidelity PCR, the polymerase is inactivated by proteinase k treatment; the proteinase k is heat inactivated and then the PCR product is digested with XhoI/EcoRI. The restriction enzymes are subsequently heat inactivated and the fragment is cloned into the corresponding sites of pREG miR30(R3-ccdB-L4) to create (E) the Entry vector pBEG R3-shRNA2-L4. F) To join several shRNAmirs in tandem within one entry vector, the recipient entry vector (from E) is digested with MluI and EcoRV and the new shRNAmir cassette is excised from its own pBEG R3-L4 Entry vector and ligated into the recipient using AscI (overlapping overhang with MluI) and EcoRV. This produces an entry vector containing two tandem shRNAmirs as shown. Alternatively, AfeI/Mlu and PmeI/Mlu can be used to daisy chain cassettes. G) The process can be reiterated indefinitely using the same enzymes as in (F) creating entry vectors with 3 or more shRNAmirs. H) Following a typical four plasmid recombination into pLEG DEST (R1–R4) to insert a B-Galactosidase gene (between attL1 and attL2 sites) followed by an internal ribosomal entry sequence (ires) to allow bicistronic expression of a puromycin resistance gene (between attR2 and attL3 sites). Directly after this there are three miRNA cassettes cloned in series between attR3 and attL4 sites and recombined together with the previous genes.
(TIF)

Figure S3 **pLEG mediated knockdown of target genes.** A) HEK 293T cells were transfected with recombinant lentiviral vectors expressing shRNAs to eGFP or dsRed or both with the pLEG-bGal-iPuro based lentiviral vectors shown in Figure 3. 10?4 cells were analyzed for GFP expression by FACS with the percentage of eGFP positive cells indicated. B) Lentiviral vectors expressing shRNAs targeting AKT2 (target sequence CGACTTCGACTATCTCAAA) or firefly luciferase (target sequence CCCGCCTGAAGTCTCTGATTAA). HEK 293T cells were infected with the viruses depicted in (B) were transfected with pCheck2-p53. C) 72 hours post-transfection firefly and Renilla luciferase were quantified and firefly luciferase activity was normalized to Renilla luciferase actively to control for transfection efficiency. D) Cell lysates were obtained in parallel and were assessed for expression of the indicated proteins by immunoblot analysis. Antisera was obtained from Cell Signalling (AKT1, cat# 2938; AKT2, cat# 3063; AKT3, cat# 8018).
(TIF)

Figure S4 **Lentiviral vectors used to transduce mouse lungs.** A) Schematic representation of pLEC Dest (R1–R2)iCreLuc, a pLEX-derived Destination vector for 2 way recombination reactions to express cDNAs transcriptionally upstream of the Cre(2a)Luc fusion. Diagram of B) pLEC eGFP iCreLuc and C) pLEx eGFP iCreLuc, which express eGFP and the Cre(2a)Luc fusion as a bicistronic transcript and differ method used to clone and sequence surrounding eGFP. (D, E) Schematic representation of integrated lentiviral vectors and function of their components.
(TIF)

Figure S5 **Control infections.** H&E stained lung tissue from A) Braf wild type mice infected with pLEG eGFP-iCreLuc or B) BrafCA/+ mice infected with pLEX eGFP-iPuro lentivirus. Note in either case, mice do not develop lung lesions.
(TIF)
