# Supplementary figures and images for: A Modular Lentiviral and Retroviral Construction System to Rapidly Generate Vectors for Gene Expression and Gene Knockdown In Vitro and In Vivo
Source: PLoS One. 2013 Oct 11;8(10):e76279. doi: 10.1371/journal.pone.0076279 (PMC3795761; doi:10.1371/journal.pone.0076279)

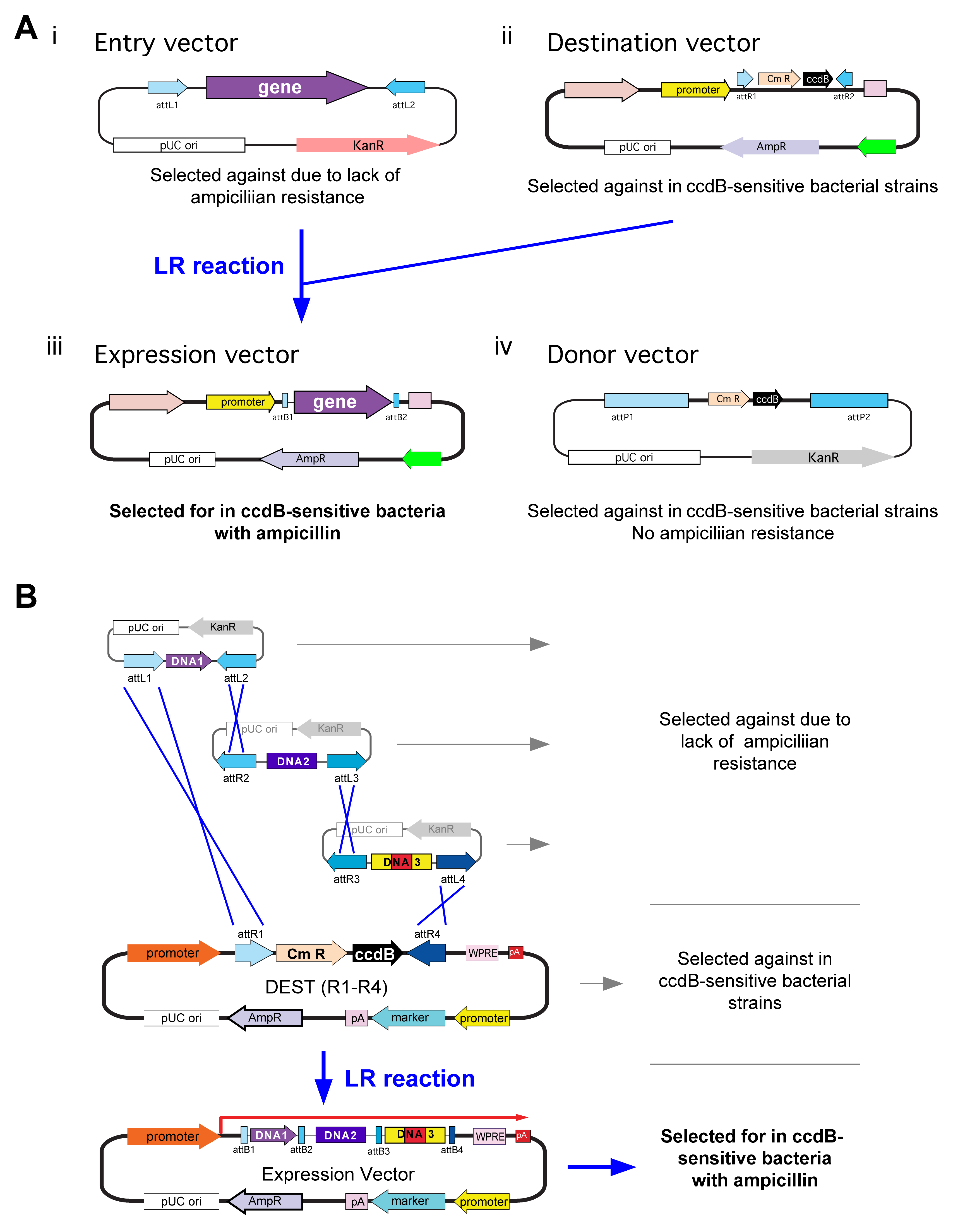

Supplement: Figure S1 — Standard and multi-plasmid Gateway recombination reactions. A) A typical “Entry vector” and “Destination vector” (i). The entry vector contains a gene insert flanked by attL1 and attL2 sites and a kanamycin resistance marker. A hypothetical Destination vector (ii) is depicted with a number of specific vector elements (non-labelled arrows and rectangles), a promoter placed upstream of a Gateway cassette with attR1 and attR2 sites flanking both chloramphenicol resistance and ccdB genes and an ampicillin resistance marker. The products of an LR reaction (iii) are an Expression vector containing all the elements of the destination vector outside the attR1 and attR2 selection cassette with the gene now flanked by attB1 and attB2 sites and a “Donor vector” (iv) containing the ccdB/chloramphenicol genes flanked by attP1/2 sites. Of the four plasmids (Entry, Destination, Donor and Expression vectors) only the Expression vector has an ampicillin resistance marker and lacks the Gateway selection cassette. B) A multi-plasmid Gateway recombination is depicted with three “Entry vectors” each conferring kanamycin resistance and having distinct DNA1, DNA2 and DNA3 flanked respectively by attL1-attL2, attR2-attL3, and attR3-attL4. These Entry vectors are selected against with ampicillin. A hypothetical Destination vector, DEST (R1–R4), compatible with a 4-way recombination is depicted. DEST (R1–R4) confers ampicillin resistance and contains the CmR/ccdB cassette. The Gateway cassette in the Destination vector is selected against in most bacteria. Following a multi-plasmid LR reaction, the reaction is transformed into ccdB-sensitive bacteria and plated on ampicillin containing plates, which selects for the Expression vector. Note: DNA1, DNA2, and DNA3 are now flanked with attB sites and are in a specific order dictated by the original sites flanking each. CmR/ccdB: Chloramphenicol resistance/ccdB cell death cassette; pUC ori: pUC origin of replication; AmpR: Ampicillin resistance ge [file pone.0076279.s001.tif]

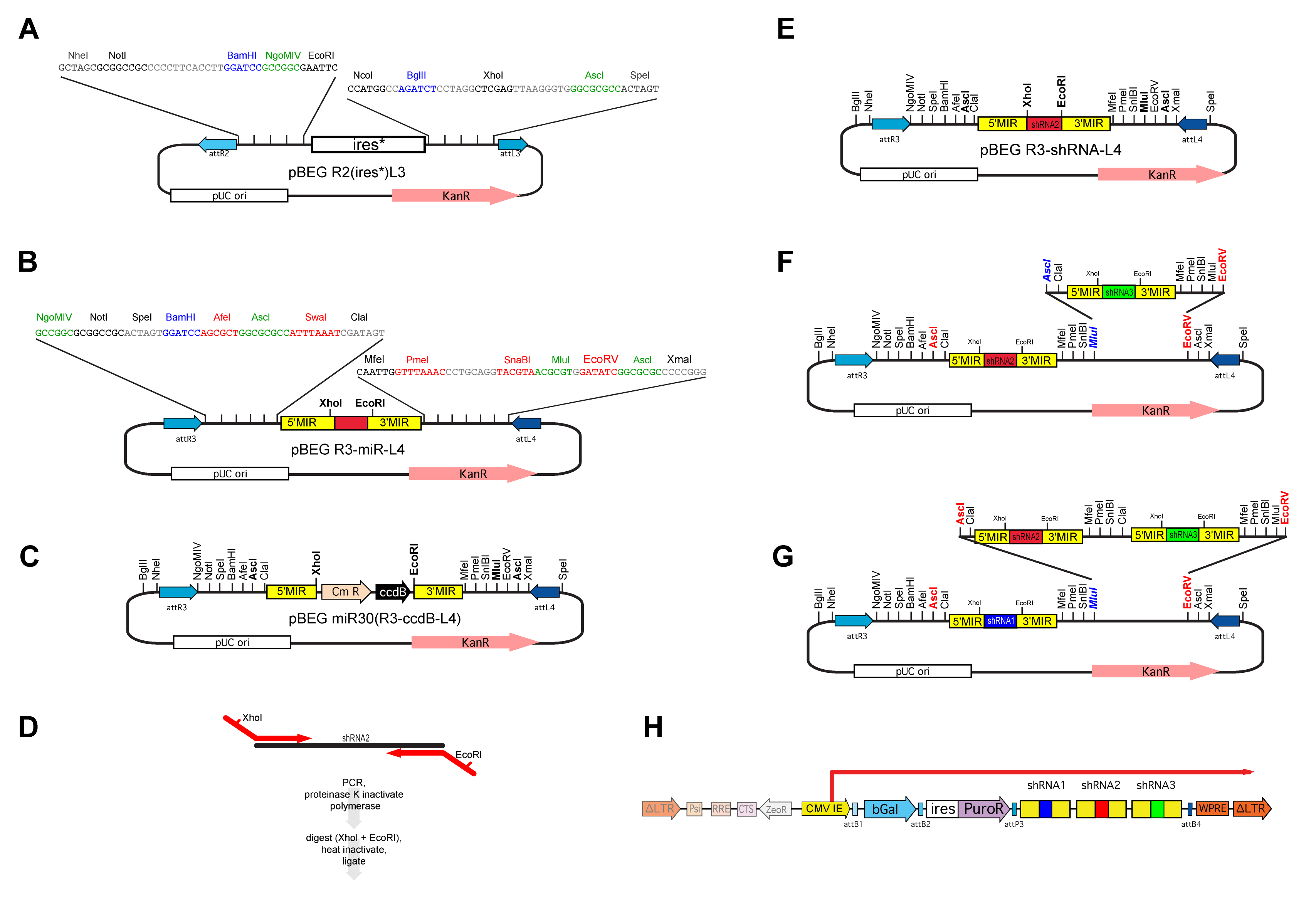

Supplement: Figure S2 — Additional Entry vectors. A) Prototypical ires-gene containing entry vector for use with 2 and 3 way recombination reactions showing a strong ires sequence (ires*) flanked by unique restriction sites for cloning up and downstream as well as attR2 and attL3 sites to allow for recombination behind an attL1-attL2 containing entry vector. B) A prototypic Entry vector containing a human miRNA-30 cassette with the shRNA sequence is cloned between unique XhoI and EcoRI sites. This cassette is flanked by a number of unique restriction enzyme sites that allow for cloning up and downstream of the cassette as well as attR3 and attL4 gateway sites to allow recombination directly after the ires-marker containing entry vector (A). C) pBEG miR30(R3-ccdB-L4) was modified to place a chloramphenicol resistance gene along with ccdB between the XhoI and EcoRI sites providing negative selection when cloning in new shRNA sequence. D) General method for the PCR amplification of novel shRNAs from a ∼100 bp oligonucleotide core (e.g. shRNA2) with two universal primers (red arrows). After high fidelity PCR, the polymerase is inactivated by proteinase k treatment; the proteinase k is heat inactivated and then the PCR product is digested with XhoI/EcoRI. The restriction enzymes are subsequently heat inactivated and the fragment is cloned into the corresponding sites of pREG miR30(R3-ccdB-L4) to create (E) the Entry vector pBEG R3-shRNA2-L4. F) To join several shRNAmirs in tandem within one entry vector, the recipient entry vector (from E) is digested with MluI and EcoRV and the new shRNAmir cassette is excised from its own pBEG R3-L4 Entry vector and ligated into the recipient using AscI (overlapping overhang with MluI) and EcoRV. This produces an entry vector containing two tandem shRNAmirs as shown. Alternatively, AfeI/Mlu and PmeI/Mlu can be used to daisy chain cassettes. G) The process can be reiterated indefinitely using the same enzymes as in (F) creating entry vectors with 3 or more shR [file pone.0076279.s002.tif]

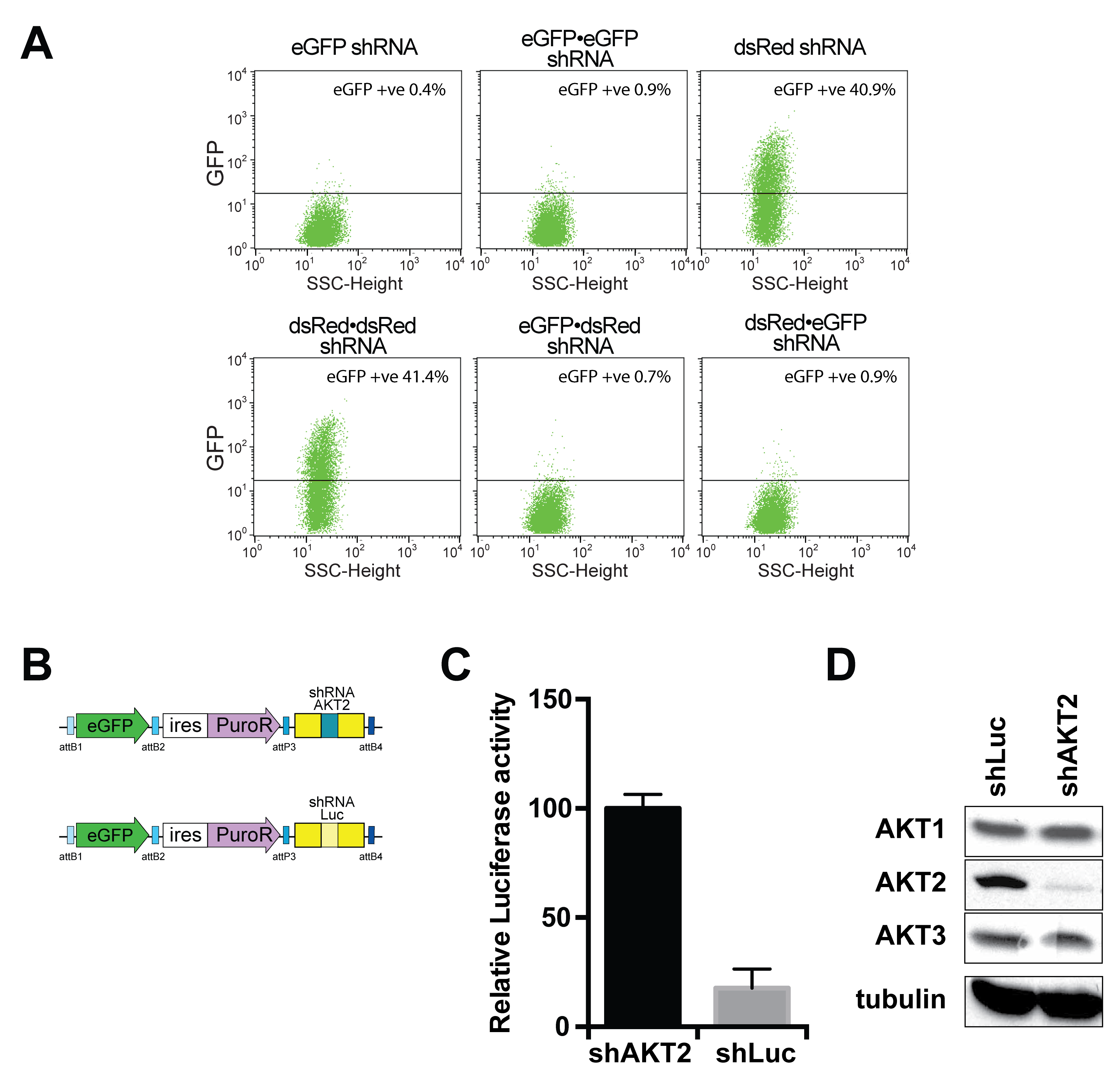

Supplement: Figure S3 — pLEG mediated knockdown of target genes. A) HEK 293T cells were transfected with recombinant lentiviral vectors expressing shRNAs to eGFP or dsRed or both with the pLEG-bGal-iPuro based lentiviral vectors shown in Figure 3. 10?4 cells were analyzed for GFP expression by FACS with the percentage of eGFP positive cells indicated. B) Lentiviral vectors expressing shRNAs targeting AKT2 (target sequence CGACTTCGACTATCTCAAA) or firefly luciferase (target sequence CCCGCCTGAAGTCTCTGATTAA). HEK 293T cells were infected with the viruses depicted in (B) were transfected with pCheck2-p53. C) 72 hours post-transfection firefly and Renilla luciferase were quantified and firefly luciferase activity was normalized to Renilla luciferase actively to control for transfection efficiency. D) Cell lysates were obtained in parallel and were assessed for expression of the indicated proteins by immunoblot analysis. Antisera was obtained from Cell Signalling (AKT1, cat# 2938; AKT2, cat# 3063; AKT3, cat# 8018). (TIF) [file pone.0076279.s003.tif]

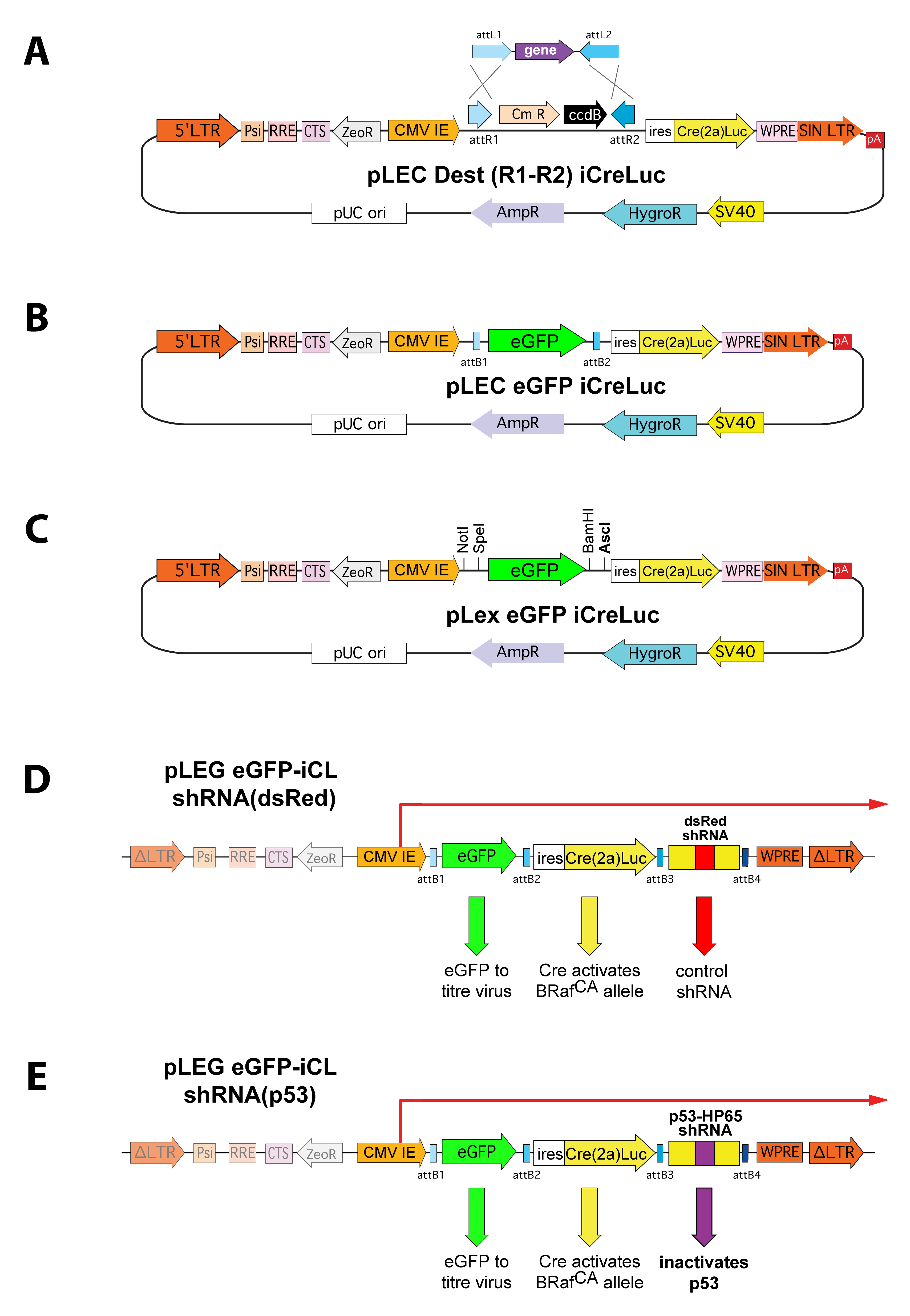

Supplement: Figure S4 — Lentiviral vectors used to transduce mouse lungs. A) Schematic representation of pLEC Dest (R1–R2)iCreLuc, a pLEX-derived Destination vector for 2 way recombination reactions to express cDNAs transcriptionally upstream of the Cre(2a)Luc fusion. Diagram of B) pLEC eGFP iCreLuc and C) pLEx eGFP iCreLuc, which express eGFP and the Cre(2a)Luc fusion as a bicistronic transcript and differ method used to clone and sequence surrounding eGFP. (D, E) Schematic representation of integrated lentiviral vectors and function of their components. (TIF) [file pone.0076279.s004.tif]

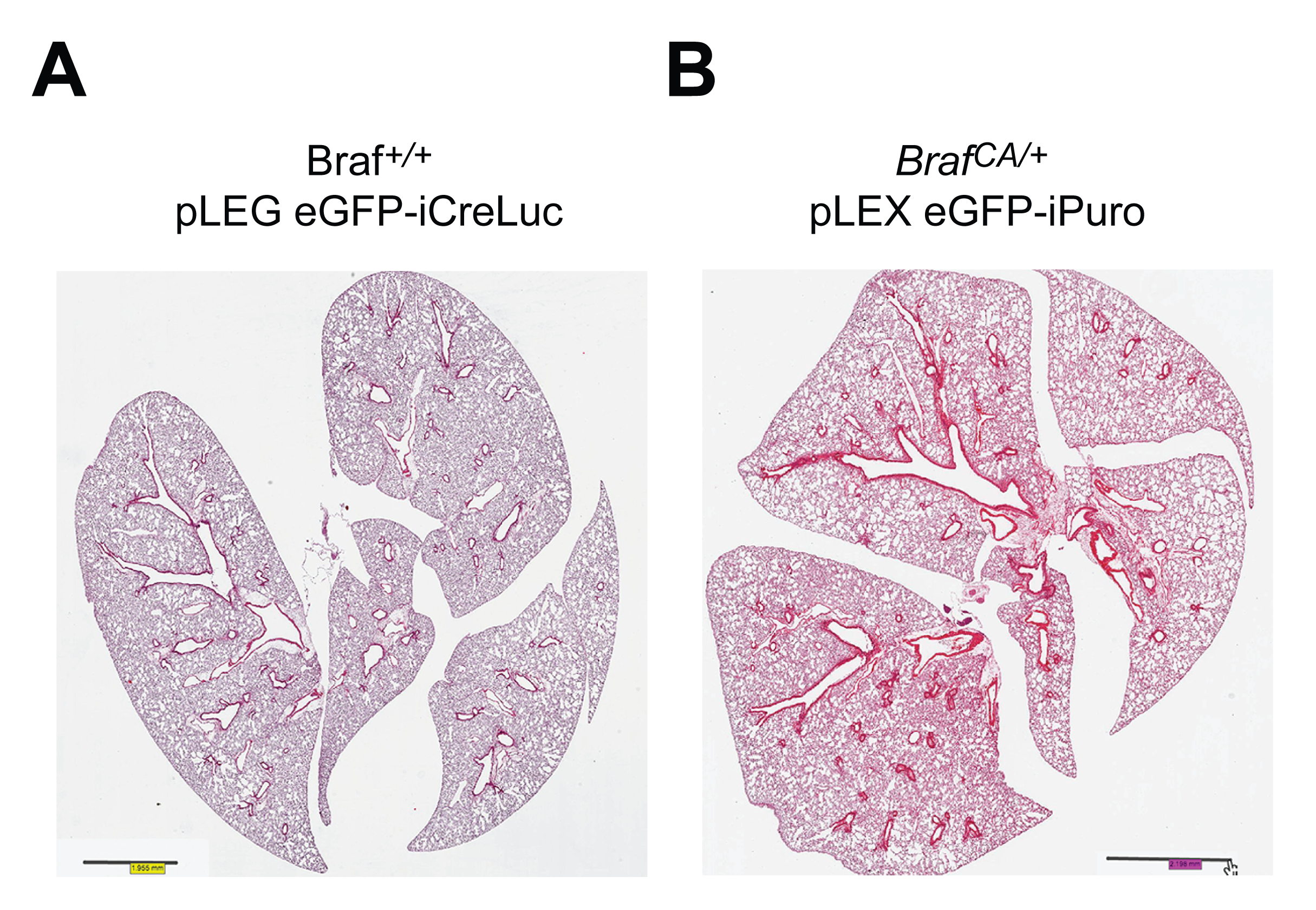

Supplement: Figure S5 — Control infections. H&E stained lung tissue from A) Braf wild type mice infected with pLEG eGFP-iCreLuc or B) BrafCA/+ mice infected with pLEX eGFP-iPuro lentivirus. Note in either case, mice do not develop lung lesions. (TIF) [file pone.0076279.s005.tif]
